# Knowledge of breast cancer among medical students in Syrian Private University, Syria: a cross-sectional study

**DOI:** 10.1186/s12909-021-02673-0

**Published:** 2021-05-01

**Authors:** Hlma Ismail, Mosa Shibani, Hanaa Wael Zahrawi, Ali Fouad Slitin, Mhd Amin Alzabibi, Fatema Mohsen, Humam Armashi, Aliaa Bakr, Khaled Turkmani, Bisher Sawaf

**Affiliations:** 1 Faculty of Medicine, Syrian Private University, Mazzeh Street, P.O. Box 36822, Damascus, Syrian Arab Republic; 2 Department of Internal Medicine, Oncology Medicine, Damascus University, Damascus, Syria; 3 AL Kalamoon General Hospital, Ministry of Health, Damascus, Syria; 4 Department of Internal Medicine, Hamad Medical Corporation, Doha, Qatar

**Keywords:** Syria, Breast Cancer, Medical students, Knowledge of risk factors

## Abstract

**Background:**

Breast cancer is the most common cancer among women and the second leading cause of cancer death globally. Since early diagnosis is crucial to reducing mortality, high levels of knowledge regarding general information, risk factors, and symptoms are required among healthcare professionals to deliver breast cancer care. This study aimed to determine Syrian medical students’ knowledge about breast cancer in the fields of general knowledge, common clinical features, and risk factors.

**Methods:**

This cross-sectional study was conducted at the Syrian Private University in October 2019 (Breast Cancer Awareness Month), Damascus, during the Syrian war crisis. Data were collected through self-administered surveys and analyzed using the Statistical Package for Social Sciences version 25.0 (SPSS Inc., United States). The chi-square test was applied to assess the relationship between the level of knowledge and gender. One way analysis of variance was performed to assess the overall differences in mean knowledge score by study year, GPA, mother’s education, and source of information. Unpaired Student’s T-test was used to analyze the differences in mean knowledge scores (continuous variable) based on smoking status and alcohol consumption.

**Results:**

Of 320 students, 301 completed the questionnaire (response rate = 94.0%), of which 179(59.5%) were males. The study revealed above-average knowledge scores (total mean = 68.4%) regarding breast cancer, general information (71.9%), common clinical features (71.6%), and risk factors (71.6%). Clinical students (4th, 5th, and 6th years) scored higher compared with pre-clinical students (1st, 2nd, and 3rd years).

**Conclusion:**

This study showed above-average knowledge scores regarding breast cancer. More efforts to correct misinformation, through reassessing the university curriculum and promoting awareness about breast cancer are required.

**Supplementary Information:**

The online version contains supplementary material available at 10.1186/s12909-021-02673-0.

## Background

Breast cancer (BC) is a significant and growing public health challenge in low and middle-income countries. Recent reports have predicted that two-thirds of new cancer cases will occur in developing countries by 2035 [1, 2]. Developing countries have limited healthcare resources, long patient waiting lists, and a shortage of screening programs. These deficiencies in resources render most populations in developing countries dependent on public healthcare systems that are ill-equipped to detect and treat BC in the early stages. Therefore, women must rely primarily on preventive measures and self-examination for risk reduction and early diagnosis of BC [3]. Early detection and prevention require deep knowledge of the risk factors, signs, and symptoms of BC, and it is the responsibility of healthcare providers to educate women about these practices [4, 5]. Unfortunately, BC awareness and the understanding of preventive practices remain poor among healthcare providers and the general public in developing countries, necessitating the need for proper awareness programs [6, 7]. Several studies revealed poor awareness of BC among medical students, non-medical students, and female residents in developing countries with knowledge levels reported to be 61.7% in Ethiopia, 42.6 and 33.4% in Saudi Arabia, 26.2% in Iran, 11.5% in Malaysia, and 4.2% in Egypt [6, 8–14]. In Syria, one study that was conducted at the Syrian Private University (SPU), the same institution in which our study takes place, indicated that only 57.5% of females students who attended the faculties of medicine, pharmacy, or dentistry had adequate knowledge about the means of early detection of breast cancer [14]. However, assessment of both male and female medical students regarding BC knowledge is required to understand the magnitude of the problem within the education system and to ensure that any intervention at the level of the community or health care system is effective.

As of 2018, BC is the most prevalent cancer among women (38.5%) and the second cause of cancer-related deaths (13.8%) after lung cancer [15]. Progress in early detection and proper treatment of BC faces many obstacles in Syria. While basic diagnostic investigations such as blood tests, biopsies, and basic imaging tools are available in all clinics across the country, BC screening programs such as mammograms are only available in Damascus. In similar fashion, magnetic resonance imaging, radiation therapy, and chemotherapy are available in major cities. Yet, they are not accessible to all patients, especially those living under siege. Finally, advanced services such as interventional radiology, genetic testing, and bone marrow transplantations are not available [16]. Additionally, as a result of the ongoing crisis in Syria, the healthcare infrastructure has sustained severe damage, with 139 attacks on health care facilities - the second-highest number of attacks worldwide - confirmed in 2018 alone. The protracted and violent nature of the conflict has destroyed of hospitals and clinics, reduced the number of health care providers, and led to severe supply shortages [17]. The paucity of the healthcare system, failed media campaigns due to demolished power plants, and the increased number of children who are not receiving education, have escalated the gaps in knowledge regarding BC among our population [17]. Al Amal association, an independent Syrian humanitarian organization committed to mitigating cancer patients’ pains, has launched BC awareness campaigns to target these knowledge gaps and provide sponsored mammograms, yet more needs to be done on a national level [18]. Finally, the majority of Syrians are Muslim, and a study shows that Islamic women neglect early BC screening due to lack of knowledge and awareness about the risk factors and manifestations of this disease [19]. This can have serious consequences as these women are only diagnosed in the advanced stages of BC. Moreover, those with poor prognosis are turned away due to limited funding, whereas those with better prognosis if treated, receive what is affordable or available. This significantly contributes to the increase in BC related deaths [2, 20].

As the future physicians who are tasked with caring for post-war generations, medical students are our soldiers in the growing battle against BC, and they are best suited to disseminate knowledge among the public. Identifying BC at an early stage is crucial for effective treatment and reducing mortality [21]. Therefore, this study aims to measure the knowledge of Syrian Private University medical students regarding BC risk factors, clinical features, and prevention methods. Our objectives are to identify the relationship between the demographic variables of these students and the knowledge of BC during this devastating crisis.

## Methods

### Study design, setting, and participants

A cross-sectional study was conducted at the faculty of medicine in the Syrian Private University (SPU) in Damascus, during Breast Cancer (BC) Awareness Month (October 2019). Ethical approval was obtained from the Institutional Review Board (IRB) of the faculty. A quantitative research approach using a self-administered validated English-language questionnaire, based on the Breast Cancer Awareness Measure (BCAM), was used in our study. This questionnaire was previously used in several published studies [8, 14, 22]. We used a convenience sampling method, and we recruited medical students who were attending a one-day breast cancer awareness conference in the Medical Faculty Library on October 25th, 2019. Questionnaires were handed out randomly to the medical students before the end of the conference proceedings. All students aged 18 years and above, who filled a consent form, were included in the study. All the responses were recorded anonymously, response to all questions was not mandatory, and students were allowed to withdraw at any time. The sample size was calculated using an online sample size calculator available at “http://www.openepi.com/SampleSize/SSPropor.htm”. After setting the maximum number of students that can be recruited in this study at 1200, the latter being the number of medical students enrolled in the medical faculty, and choosing the confidence level and confidence interval to be 95 and 5%, respectively, the calculator generated a target sample size of 291 students. The conference was attended by 320 medical students. They all provided their consent to participate in the study, and they were handed a copy of the questionnaire to fill out. The questionnaire included 50 questions divided into five sections: socio-demographic information (9 questions), including academic year, marital status, gender, current residence, grade point average (GPA), smoking, alcohol consumption, and mother’s educational level; BC general knowledge (16 questions); signs and symptoms (13 questions); risk factors (11 questions); source of knowledge (1 question). The questionnaire is available in Additional file 1.

### Statistical analysis

The number of questions related to BC knowledge was 40. A scoring system was used to analyze the students’ knowledge, where a score of “1” was given for a correct answer and a score of “0” was given for an incorrect answer. The mean percentage score for knowledge was calculated as follows: (sum of scores obtained/maximum scores that could be obtained) × 100. Students’ total mean knowledge score in all the subsections, and mean knowledge score of each subsection were calculated and used to categorize students’ scores as either low (0–24.9%), below average (25–49.9%), above-average (50–74.9%) or high (75–100%) [23]. Statistical Package for Social Sciences (SPSS) version 25.0 was used for data analysis. Continuous data was reported as mean and standard deviation (SD) and categorical data was described as frequencies and percentages. Pearson Chi-square test was applied to assess the relationship between the level of knowledge and gender. One-way analysis of variance (ANOVA) was performed to assess the overall differences in mean knowledge scores (continuous variables) by study year, GPA, mother’s education, and source of information (categorical variables). Finally, unpaired Student T-test was used to analyze the differences in mean knowledge scores (continuous variable) based on smoking status and alcohol consumption. The statistical significance was set at *p* < 0.05.

## Results

### Socio-demographic characteristics

Of the 320 students who were handed the questionnaire, 301 students completed the questionnaire, 11 students submitted an incomplete questionnaire, and 8 students withdrew their consent to participate in the study. As we only included complete questionnaires in this study, our final sample size was 301 medical students placing the response rate at 94.0%. Of these, 179(59.5%) were males, and 122(40.5%) were females. Fifth-year students represented the majority of responders [87(28.9%)], while second-year students represented the minority [10(3.3%)]. Moreover, most students were single [266(88.4%)] and living in Damascus [274(91%)]. Finally, 105(34.9%), and 28(9.3%) students were smokers and alcohol consumers, respectively, and female students accounted for 20.5% of the smokers and 1.63% of the alcohol consumers (Table 1).

**Table 1:** Socio-demographic characteristics of participants: (*n* = 301)

			**Mother’s Education**	Primary	19 (6.3%)
				Secondary	24 (8%)
				High School	95 (31.6%)
**Gender**	Male	179 (59.5%)		University	126 (41.9%)
	Female	122 (40.5%)		Postgraduate	37 (12.3%)
**Social Status**	Single	266 (88.4%)	**Current residence**	Urban	274 (91%)
	In a relationship	28 (9.3%)		Rural	27 (9%)
	Married	7 (2.3%)	**College Year**	1st	31 (10.3%)
**GPA**	< 2.0	34 (12.5%)		2nd	10 (3.3%)
	2.0–2.5	164 (60.1%)		3rd	52 (17.3%)
	2.5–3.0	59 (21.6%)		4th	55 (18.3%)
	> 3.0	16 (5.9%)		5th	87 (28.9%)
**Alcohol use**	yes	28 (9.3%)		6th	66 (21.9%)
	no	273 (90.7%)	**smoking**	yes	105 (34.9%)
				no	196 (65.1%)

### Breast cancer in general

**Fig. 1:**
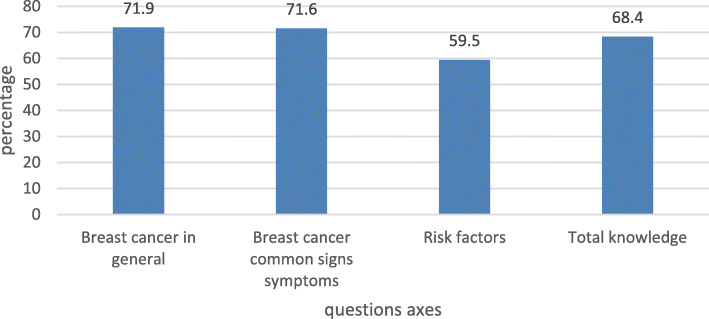
Percentages of knowledge in each section

The students’ total mean knowledge regarding BC was above-average (68.4%), and the level of knowledge in the general information section was also above-average (71.9%) (Fig. 1). The majority knew that the most common BC metastasis site is the axillary lymph nodes [291(96.7%)]. Moreover, 273 students (90.7%) knew about the BC screening methods, and 132 students (43.9%) were unaware of mammograms, of which 50.0% of the latter were females. Finally, 286 students (95.0%) believed that all women aged 40 years or above need to have a mammogram at least every 2 years, and 237 students (78.7%) were of the view that women aged 50 years or above are more likely to have BC than younger women.

When asked about the factors that contribute to increased incidence of BC, 176 students (58.5%) thought that stress contributes to BC, and 278 students (92.4%) correctly answered that family history has a role in increasing BC incidence. Moreover, 151 students (50.2%) students thought that the increase in breast size is not an indicator of increased likelihood of BC occurrence. Finally, 89 students (29.6%) students supposed that parental breastfeeding can be a factor, and 182 students (60.5%) thought that wearing a tight bra will increase the incidence (Table 2).

**Table 2:** General knowledge regarding breast cancer (*n* = 301)

			T (*N* = 301)	F (*N* = 122)	M (*N* = 179)	Chi-square	*P*-value
Do you know any breast cancer screening methods?	No	N (%)	28 (9.3)	7 (5.7)	21 (11.7)	3.0897	.078
	Yes	N (%)	273 (90.7)	115 (94.3)	158 (88.3%)		
Do you know what mammograms are?	No	N (%)	132 (43.9)	61 (50.0)	71 (39.7)	3.1474	.076
	Yes	N (%)	169 (56.1)	61 (50.0)	108 (60.3)		
Every woman over the age of 40 needs to have a mammogram at least every two years	No	N (%)	15 (5.0)	6 (4.9)	9 (5.0)	0.0019	.965
	Yes	N (%)	286 (95.0)	116 (95.1)	170 (95.0)		
One in eight women will develop breast cancer at one point in their lives	No	N (%)	79 (95.0)	35 (28.7)	44 (24.6)	0.6324	.426
	Yes	N (%)	222 (73.8)	87 (71.3)	135 (75.4)		
Women at the age of 50 are more likely to have breast cancer than young women	No	N (%)	64 (21.3)	25 (20.5)	39 (21.8)	0.0728	.787
	Yes	N (%)	237 (78.7)	97 (79.5)	140 (78.2)		
Stress has been shown to contribute to breast cancer	No	N (%)	125 (41.5)	44 (36.1)	81 (45.3)	2.5211	.112
	Yes	N (%)	176 (58.5)	78 (63.9)	98 (54.7)		
Family history has a role in increasing the incidence	No	N (%)	23 (7.6)	8 (6.6)	15 (8.4)	0.3415	.558
	Yes	N (%)	278 (92.4)	114 (93.4)	164(91.6)		
The incidence of breast cancer and ovarian cancer in the same family is an indicator of a genetic basis of disease	No	N (%)	35 (11.6)	13 (10.7)	22(12.3)	0.1887	.664
	Yes	N (%)	266 (88.4)	109 (89.3)	157 (87.7)		
Increasing in the breast size is an indicator of the likelihood of incidence	No	N (%)	151 (50.2)	67 (54.9)	84 (46.9)	1.853	.173
	Yes	N (%)	150 (49.8)	55 (45.1)	95 (53.1)		
Is parental breastfeeding related to the possibility of incidence	No	N (%)	212 (70.4)	89 (73.0)	123 (68.7)	0.625	.429
	Yes	N (%)	89 (29.6)	33 (27.0)	56 (31.3)		
Is exercise related to the possibility of incidence	No	N (%)	251 (83.4)	101 (82.8)	150 (83.8)	0.0536	.816
	Yes	N (%)	50 (16.6)	21 (17.2)	29 (16.2)		
Chemotherapy is always the provided treatment	No	N (%)	217 (72.1)	90 (73.8)	127 (70.9)	0.2869	.592
	Yes	N (%)	84 (27.9)	32 (26.2)	52 (29.1)		
Is there a relationship between the development of cancer and trauma to the breast	No	N (%)	144 (47.8)	50 (41.0)	94 (52.5)	3.8655	.049
	Yes	N (%)	157 (52.2)	72 (59.0)	85 (59.0)		
Does trauma to the breast during intercourse have a role in the occurrence of cancer	No	N (%)	209 (69.4)	82 (67.2)	127 (70.9)	0.4773	.489
	Yes	N (%)	92 (30.6)	40 (32.8)	52 (29.1)		
Can a tight bra increase the incidence of breast cancer?	No	N (%)	119 (39.5)	47 (38.5)	72 (40.2)	0.0876	.767
	Yes	N (%)	182 (60.5)	75 (61.5)	107 (59.8)		
What is the most commonplace for proximal breast cancer metastases?	Armpit	N (%)	291 (96.7)	118 (96.7)	173 (96.6)	0.0012	.972
	neck	N (%)	9 (3.0)	3 (2.5)	6 (3.4)	−0.447	0.655
	Supraclavicular	N (%)	1 (0.3)	1 (0.8)	0(0.0)	−0.827	0.408

**Fig. 2:**
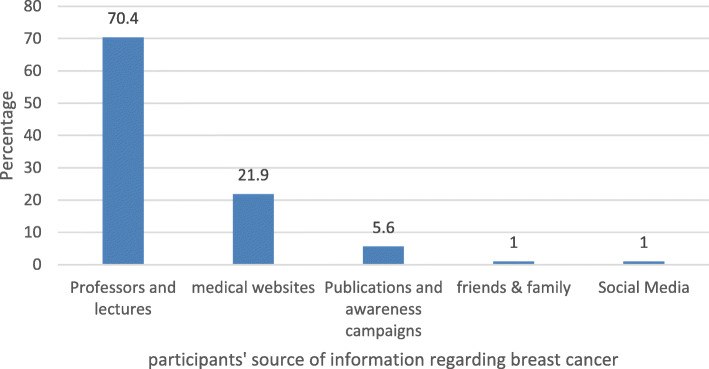
Sources of information

The majority of responders cited professors and lectures as their main source of information [212(70.4%)]. On the other hand, only 1% of the students considered both family and friends, and social media as their main sources of information (Fig. 2).

### Breast cancer common signs and symptoms

Regarding the common symptoms of BC, the level of knowledge was above-average (71.6%) (Fig. 1). Most students identified a mass in the breast [298(99.0%)] or a mass in the axillary region [277(92.0%)] as the most known BC symptoms. The responders also selected changes in pigmentation [264(87.7%)], nipple discharge [275(91.4%)], nipple retraction [268(89.0%)], change in the symmetry [274(91.0%)], ulcers [215(71.4%)], and scaliness of the skin [216(71.8%)] as common signs and symptoms of BC. However, only 118 students (39.2%) identified rash as a common BC sign. Misconceptions were also noted where 226 students (75.1%) and 200 students (66.4%) named pain in the nipple and breast, and pain in the axillary region as common symptoms of BC (Table 3).

**Table 3:** Knowledge regarding common clinical features of breast cancer (*n* = 301)

			T (*N* = 301)	F (*N* = 122)	M (*N* = 179)	Chi-square	*P*-value
Feeling a mass in the breast	No	N (%)	3 (1.0)	1 (0.8)	2 (1.1)	0.0651	.798
	Yes	N (%)	298 (99.0)	121 (99.2)	177 (98.9)		
Feeling a mass in the armpit	No	N (%)	24 (8.0)	6 (4.9)	18 (10.1)	2.61	.106
	Yes	N (%)	277 (92.0)	116 (95.1)	161 (89.9)		
Changes in pigmentation	No	N (%)	37 (12.3)	16 (13.1)	21 (11.7)	0.1287	.719
	Yes	N (%)	264 (87.7)	106 (86.9)	158 (88.3)		
Nipple discharge (other than breast milk)	No	N (%)	26 (8.6)	7 (5.7)	19 (10.6)	2.1865	.139
	Yes	N (%)	275 (91.4)	115 (94.3)	160 (89.4)		
Nipple retraction (turning inward)	No	N (%)	33 (11.0)	11 (9.0)	22 (12.3)	0.7967	.372
	Yes	N (%)	268 (89.0)	111 (91.0)	157 (87.7)		
Change in nipple position	No	N (%)	76 (25.2)	25 (20.7)	51 (28.5)	2.3403	.126
	Yes	N (%)	224 (74.4)	96 (79.3)	128 (71.5)		
Change in breast symmetry	No	N (%)	27 (9.0)	12 (9.8)	15 (8.4)	0.1884	.664
	Yes	N (%)	274 (91.0)	110 (90.2)	164 (91.6)		
Ulcers	No	N (%)	86 (28.6)	27 (22.1)	59 (33.0)	4.1695	.041
	Yes	N (%)	215 (71.4)	95 (77.9)	120 (67.0)		
Scaling or thickening of the nipple or breast skin	No	N (%)	85 (28.2)	32 (26.2)	53 (29.6)	0.4089	.522
	Yes	N (%)	216 (71.8)	90 (73.8)	126 (70.4)		
Redness of the nipple or breast skin.	No	N (%)	104 (34.6)	38 (31.1)	66 (36.9)	1.0512	.305
	Yes	N (%)	197 (65.4)	84 (68.9)	113(68.9)		
Skin rash on the breast	No	N (%)	183 (60.8)	75 (61.5)	108 (60.3)	0.0396	.842
	Yes	N (%)	118 (39.2)	47 (38.5)	71 (39.7)		
Pain in the armpit	No	N (%)	101 (33.6)	30 (24.6)	71 (39.7)	7.3947	.006
	Yes	N (%)	200 (66.4)	92 (75.4)	108 (60.3)		
Nipple or breast pain	No	N (%)	75 (24.9)	24 (19.7)	51(28.5)	3.0165	.082
	Yes	N (%)	226 (75.1)	98 (80.3)	128 (71.5)		

### Knowledge regarding risk factors

Our results revealed that the responders had above-average knowledge (59.5%) about the risk factors of BC (59.5%), which was recognizably lower than other sections (Fig. 1). The risk factors of BC that were most commonly named by the students were postmenopausal estrogens [249(82.7%)], aging [241(80.1%)], and oral contraceptives [229(76.1%)]. Other selected risk factors included smoking [189(62.8%)], history of benign tumors [186(61.8%)], late menopause [178(59.1%)], early puberty [161(53.5%)], and weight gain (BMI > 25) [161(53.5%)]. Same with BC signs and symptoms, misconceptions were also noted in this section as 212 students (70.4%) and 161 students (53.3%) named history of breast inflammatory disease and history of breast trauma, respectively, as risk factors of BC (Table 4).

**Table 4:** Knowledge regarding risk factors of breast cancer (*n* = 301)

			T (*N* = 301)	F (*N* = 122)	M (*N* = 179)	Chi-square	*P*-value
**Early puberty**	No	N (%)	140 (46.5)	55 (45.1)	85 (47.5)	0.1685	.681
	Yes	N (%)	161 (53.5)	67 (54.9)	94 (52.5)		
**Late menopause**	No	N (%)	123 (40.9)	57 (46.7)	66 (36.9)	2.9128	.087
	Yes	N (%)	178 (59.1)	65 (53.3)	113 (63.1)		
**Oral contraceptives**	No	N (%)	72 (23.9)	28 (23.0)	44 (24.6)	0.1059	.744
	Yes	N (%)	229 (76.1)	94 (77.0)	135 (75.4)		
**Postmenopausal estrogens**	No	N (%)	52 (17.3)	20(16.4)	32(17.9)	0.1117	.738
	Yes	N (%)	249 (82.7)	102 (83.6)	147 (82.1)		
**history of trauma on the breast**	No	N (%)	140 (46.5)	52 (42.6)	88 (49.2)	1.247	.264
	Yes	N (%)	161 (53.5)	70 (57.4)	91 (50.8)		
**History of benign tumors**	No	N (%)	115(38.2)	44 (36.1)	71 (39.7)	0.3981	.528
	Yes	N (%)	186 (61.8)	78 (63.9)	108 (60.3)		
**History of Inflammatory disease of the breast**	No	N (%)	89 (29.6)	41 (33.6)	48 (26.8)	1.6066	.204
	Yes	N (%)	212 (70.4)	81 (66.4)	131 (73.2)		
**Alcoholic**	No	N (%)	170 (56.5)	70 (57.4)	100 (55.9)	0.0674	.795
	Yes	N (%)	131 (43.5)	52 (42.6)	79 (44.1)		
**Smoking**	No	N (%)	112 (37.2)	48 (39.3)	64 (35.8)	0.4002	.526
	Yes	N (%)	189 (62.8)	74 (60.7)	115 (64.2)		
**Aging**	No	N (%)	60 (19.9)	25 (20.5)	35 (19.6)	0.0401	.841
	Yes	N (%)	241 (80.1)	97 (79.5)	144 (80.4)		
**Weight gain (BMI > 25)**	No	N (%)	140 (46.5)	50 (41.0)	90 (50.3)	2.5199	.112
	Yes	N (%)	161 (53.5)	72 (59.0)	89 (49.7)		

### Correlation between variables

One-way ANOVA was used to study the relationship between the overall mean knowledge and students’ demographic characteristics. Surprisingly, students with lower GPA (2–2.5) had a mean knowledge of 69.7%, while students with higher GPA of 2.5–3 and >  3 had a mean knowledge of 69.4 and 67.1%, respectively (F = 2.979, *p*-value = 0.032*).

Data showed a lower knowledge level among students whose mothers only received primary education (64.4%) compared with those whose mothers have a professional degree (72.2%) (F = 2.448, *p*-value = 0.046*). Finally, unpaired Student’s T-test showed a slightly significant difference in the overall mean knowledge scores between smokers and non-smokers (69.99% vs. 67.60%) (T = − 2.016, *p*-value = 0.045), and consumers and non-consumers of alcohol (72.0% vs. 68%) (T = − 2.032, *p* = 0.043) (Table 5).

**Table 5:** Relationship between mean knowledge scores and GPA, mothers’ education, smoking, and consuming alcohol

	Descriptive analysis								One Way ANOVA Test	
		N	Mean	Std. Deviation	95% Confidence Interval for Mean		Min	Max	F	*p*.value
					Lower Bound	Upper Bound				
GPA	< 2	34	64.5	9.4	61.2	67.8	47.6	83.3	2.979	0.032*
	2–2.5	164	69.74	9.4	68.3	71.2	40.5	90.5		
	2.5–3	59	69.37	10.4	66.7	72.1	42.9	90.5		
	> = 3	16	67.07	10.7	61.3	72.8	50	83.3		
	total	273	68.85	9.8	67.7	70	40.5	90.5		
Mother’s Education	primary	19	64.41	12.7	58.3	70.5	42.9	81	2.448	0.046*
	secondary	24	68.13	10.3	63.8	72.5	47.6	88.1		
	high school	95	67.47	9.8	65.5	69.5	42.9	90.5		
	university	126	68.73	9.6	67	70.4	40.5	85.7		
	post-graduate	37	72.2	8.1	69.5	74.9	50	85.7		
	total	301	68.44	9.9	67.3	69.6	40.5	90.5		
									T	p.value
Smoking	no	196	67.60	10.1	66.1	69.0	40.4	90.4	−2.016	0.045*
	yes	105	69.99	9.2	68.2	71.7	42.8	90.4		
	Total	301	68.45	9.85	67.3	69.5	40.4	90.4		
Alcohol consumption	no	273	68.0	9.96	66.8	69.2	40.4	90.4	−2.032	0.043*
	yes	28	72.0	8.08	68.8	75.1	47.6	83.3		
	Total	301	68.4	9.85	67.3	69.5	40.4	90.4		

**Fig. 3:**
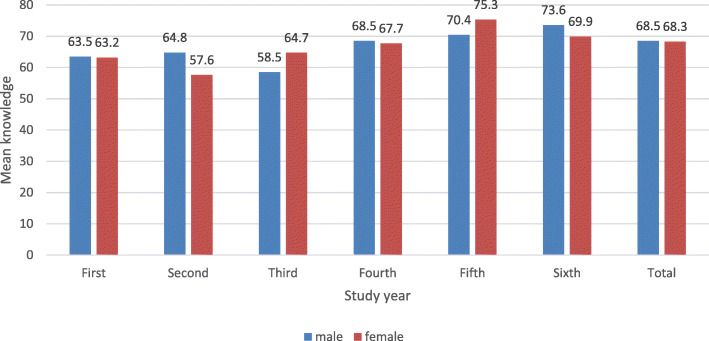
Comparison between the year of study and total knowledge in both genders

The students who showed above-average knowledge about BC chose professors and lectures (69.6%), medical websites (67.5%), and friends and family (58.7%) as their sources of information. There was no significant difference in the overall knowledge between males and females (Table 6). First-year (male 63.5%, female 63.2%), second-year (male 64.8%, female 57.6%), third-year (male 58.5%, female 64.7%), fourth-year (male 68.5%, female 67.7%), and sixth year (male 73.6%, female 69.9%) students showed above-average knowledge scores, whereas fifth-year female students had high knowledge (75.3%) compared with above-average knowledge among fifth-year male students (70.4%) (Fig. 3).

**Table 6:** Relationship between mean knowledge score and gender

Group Statistics						T test		
		Gender	N	Mean (%)	Std. Deviation	Mean Difference	T test value	*p*.value
Mean Knowledge	Gender	Males	179	68.5	10.3	0.229	0.197	0.844
		Females	122	68.3	9.2

**Fig. 4:**
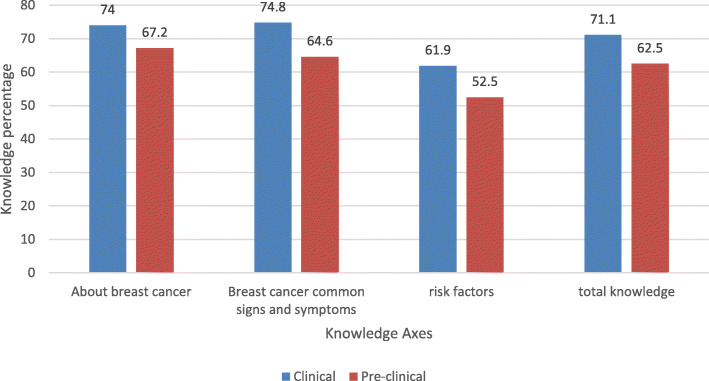
Comparison between clinical and preclinical years of knowledge

The total mean knowledge score was above-average among both the clinical students (71.1%) and the preclinical students (62.5%). Both clinical and preclinical students showed above-average knowledge about the risk factors (52.5 and 61.9%). However, their levels of knowledge in this field were lower than what they exhibited in other sections of the questionnaire (Fig. 4).

## Discussion

No significant difference in the level of BC knowledge was found between males and females, smokers and non-smokers, and consumers and non-consumers of alcohol. These results are expected, as the sample in our study consists of medical students who receive information equally regardless of their gender or daily habits. Our findings on gender were consistent with previous studies performed on similar populations in the region [9, 24, 25]. However, we could not find any previous studies that discussed the effects of smoking or alcohol consumption on breast cancer awareness, or medical knowledge in general, among medical students. On another note, even though the majority of responders were fifth-year students, this was not reflective of a higher willingness to participate in this survey compared to other groups such as second-year students. The attendance of the conference in which the participants were recruited was open to all students from all medical years. Therefore, most responders were fifth-year medical students, because they constituted the majority of the conference attendants. As a result, it was not possible for us to determine which group of students had the highest proclivity to partake in this survey.

Most of our students were aware of BC screening methods, and their level of knowledge in this field was significantly higher than that reported in an Egyptian study [25]. Moreover, our female students showed a higher level of knowledge about BC screening methods compared to Malaysian and Saudi female medical students [10, 11]. However, the fact that 43.9% of our responders were unaware of mammography as a BC screening method sheds light on the knowledge gap in this field among medical students and emphasizes the importance of incorporating this information early and clearly in the curriculum. BC screening mammography is limited to the capital city, Damascus. Hence, our university should cooperate with clinics that readily have these diagnostic tools to allow our students to be made aware of them and to learn about them and their scope of work [16].

Even though mammography is one of the cheapest and most efficient screening tools for early detection, it is only helpful if done regularly. The majority of our students knew that every woman over the age of 40 needs to have a mammogram at least once every 2 years. The level of knowledge of our students in this topic was significantly higher than those reported by similar studies that took place in Egypt and Colombia [25, 26]. In similar fashion, 95.1% of our female students agreed with the need for regular screening, whereas only 67.8% of Pakistani female medical students were aware of this information [27].

Studies show that approximately 1 in 8 women (13%) will be diagnosed with invasive BC [28], and our students were well aware of this information. Women diagnosed with BC frequently attribute this disease to psychological stress, even though a link between stress or challenging life events and an increased BC risk has yet to be established [29]. Such misconception was also prevalent among our students as 58.5% of the responders believed that psychological stress is associated with developing BC. Similar results were also reported among Chinese and Egyptian medical students [12, 30]. Regarding BC preventive measures, breastfeeding has been shown to not only reduce the incidence of BC but also to provide other benefits to mothers such as reduced risk of endometrial and ovarian cancers [31, 32]. The data showed that our students agreed that breastfeeding does not affect the incidence of BC. Similar results were noted among the medical students of Ethiopia, Pakistan, Angola, and Egypt [6, 12, 24, 27, 33]. Studies conducted in Saudi Arabia in the cities of Makkah and Taif reported that 63.0 and 80% of medical students, respectively, identified that refraining from breastfeeding may increase the incidence of BC [11, 13]. Studies indicate that physical activity and regular exercise have beneficial physiological effects that can contribute to the prevention of BC [32]. The majority of our responders knew that exercise is not a risk factor for BC; a result which was in accordance with what was reported in other studies [24, 33].

Our students as identified professors and lectures as their main sources of information on BC, which was similar to study on Saudi medical students [11]. Our results also showed that these students had the highest level of knowledge. These findings underscore the significant role of university education in enhancing general knowledge. In contrast, medical students in other studies named the media, medical websites, and self-learning as their primary sources of information on BC [11, 25]. Being knowledgeable about BC clinical features and being able to report any noticeable abnormalities to healthcare providers, is known as “breast awareness”. This self-awareness can help in early detection of BC and thereby reduce mortality rates [34]. Common symptoms of BC include changes in appearance or texture of the breast or nipple, nipple discharge, erythema, and scars [35]. In our study, the overall knowledge of signs and symptoms was higher among clinical year students compared with preclinical years. Mass in the breast is the most common symptom of BC, and it is noted in around 83% of BC patients [36]. Almost all our students were aware of this symptom, and this result is significantly higher than what is reported in other studies [6, 10, 11, 13, 33]. Such a high level of knowledge may be attributed to mass media campaigns serving as an effective means of knowledge dissemination by targeting a heterogeneous audience [37, 38].

There is a widespread misconception that BC is associated with pain, when pain is in fact an infrequent early sign of BC and occurs in only 6% of patients [36]. Our results showed that the majority of students thought that pain in the breast, axillary region, or nipples is a common symptom of BC. The regular reporting of this misconception by studies similar to ours is an indication of its prevalence among medical students in developing countries [11, 24, 33]. Several factors affect an individual’s risk of developing BC, some of which are modifiable and largely related to lifestyle such as smoking and alcohol consumption, in addition to other economic, social, and environmental factors [4]. Women must know these factors early in life so they can make the right lifestyle choices, as increasing women’s awareness can change their risk perceptions and behaviors [39]. On the other hand, other risk factors such as family history of BC are unmodifiable. Our study revealed insufficient knowledge among the responders regarding risk factors that are associated with BC, and family history was recognized by a fewer number of our students as a risk factor of BC compared with other studies [6, 9–13, 33].

BC incidence is unequivocally related to age, with rates increasing proportionally with age. Older women are six times more at risk to develop BC and have an eight-fold higher mortality rate compared with younger women [40]. The most commonly named risk factors in our study were postmenopausal estrogens, old age, and oral contraceptives, which was in accordance with other studies [27, 33]. On the other hand, misconceptions about early puberty (age < 12 years) and late menopause (age > 55 years) being associated with an increased risk of BC due to longer periods of hormonal exposure, were observed amongst our students. In similar fashion, most published studies have demonstrated diminished knowledge about BC risk factors among medical students [6, 10, 11, 13, 14, 33]. Knowledge regarding risk factors and risk-reducing strategies is important for primary prevention of BC and raising awareness on them among our students must be in the core of the medical curriculum. A well-planned targeted approach to address the misconceptions regarding BC by our university is pivotal, and it requires specialist teaching and input from BC awareness programs. Small associations such as Al Amal have launched BC awareness campaigns to target these knowledge gaps in certain regions, but more needs to be done on a national level [18].

Studies show that some daily habits, such as smoking, increase BC risk in women, especially in those who start smoking at younger ages [41]. Moreover, the International Agency for Research on Cancer estimates a 4–15% increase in BC risk for women who consume even little amounts of alcohol (≤1 drink = 12.5 g/day) [42, 43]. Our students showed lack of knowledge regarding smoking and alcohol as risk factors. Moreover, our results revealed a higher percentage of female students who are smokers than what was reported in one study conducted on students in Damascus University [44], and other studies conducted on medical students in Dublin and Brazil [45, 46]. Our study showed that few female students were alcohol consumers. However, the prevalence of alcohol drinking among our female students was lower than what was reported about medical students in Greece and Thailand [47, 48]. These future doctors play a pivotal role in disseminating awareness among the population about the perilous effects of smoking and alcohol consumption. Doctors should therefore embrace their duties by behaving as role models for our society.

### Limitations

The elements of limitation recognized within this study include: 1. Sample population, derived from only one faculty 2. This study does not reflect the overall Syrian medical students 3. Trusted published data regarding the impact of the Syrian crisis on the educational system was not found. To overcome these limitations, a further study on a national level is required.

## Conclusion

In conclusion, this study revealed an above-average level of knowledge regarding BC. Since the level of knowledge of the risk factors was lower than that of other sections, it is necessary to address this gap on an educational level by modifying medical programs to include extensive training in the areas of BC preventive measures and early diagnosis.. Further evaluation of the methods of teaching, input from medical boards, and curriculum advisors will produce doctors who have sufficient knowledge about BC. Our study provides useful results that can help in the planning of health education campaigns aimed at correcting misinformation and promoting the use of mammograms for early diagnosis of BC among women in the country.

## Supplementary Information

**Additional file 1.** The study questionnaire.

## Data Availability

All data related to this paper’s conclusion are available and stored by the authors. All data are available from the corresponding author on a reasonable request.

## Abbreviations

BC: Breast Cancer
BRCA1 gene: Breast Cancer gene1
BRCA2 gene: Breast Cancer gene2
SPU: Syrian Private University
IRB: Institutional Review Board
GPA: Grade Point Average
SD: Standard Deviations
ANOVA: Analysis Of Variance
SPSS: Statistical Package for Social Sciences
BMI: Body Mass Index
OCP: Oral Contraceptive Pills
HRT: Hormone Replacement Therapy
