# Supplementary material for: Knowledge of breast cancer among medical students in Syrian Private University, Syria: a cross-sectional study
Source: BMC Med Educ. 2021 May 1;21:251. doi: 10.1186/s12909-021-02673-0 (PMC8088684; doi:10.1186/s12909-021-02673-0)
Supplement: Supplementary file 1 — Additional file 1. The study questionnaire. [file 12909_2021_2673_MOESM1_ESM.docx]

# **
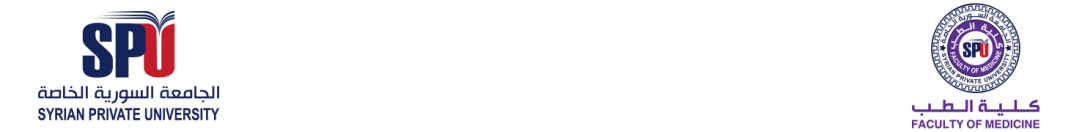
Appendix 1.**

**Ethical approval of the questionnaire was obtained from the Institutional Review Board (IRB), Faculty of Medicine.**

| **Socio-demographic Characteristics** | |
| --- | --- |
|  | **Alcohol consuming** **:**  □Yes □No |
| **Gender:** □Male □Female | **Current residence:** □City □Rural |
| **Social Status:** □Single □In a relationship □Married | **GPA:** □< 2.0 □2.0 - 2.5 □2.5 - 3.0 □> 3.0 |
| **Smoking :**  □Yes □No | **Alcohol:**  □Yes □No |
| **College Year:** □1st □2nd □3rd □4th □5th □6th | **Mother's Education:**  □Primary □Secondary □High School □University □Post Graduate |

| **Breast cancer in general** | | |
| --- | --- | --- |
| **No** | **Yes** |  |
|  |  | Do you know any breast cancer screening methods? |
|  |  |  |
|  |  | Do you know what mammograms are? |
|  |  |  |
|  |  | Every woman over the age of 40 needs to have a mammogram at least every two years |
|  |  |  |
|  |  | One in eight women will develop breast cancer at one point in their lives |
|  |  |  |
|  |  | Women at the age of 50 are more likely to have breast cancer than young women |
|  |  |  |
|  |  | Stress has been shown to contribute to breast cancer |
|  |  |  |
|  |  | Family history has a role in increasing the incidence |
|  |  |  |
|  |  | The incidence of breast cancer and ovarian cancer in the same family is an indicator of a genetic basis of disease |
|  |  |  |
|  |  | Increasing in the breast size is an indicator of the likelihood of incidence |
|  |  |  |
|  |  | Is parental breastfeeding related to the possibility of incidence |
|  |  |  |
|  |  | Is exercise related to the possibility of incidence |
|  |  |  |
|  |  | Chemotherapy is always the provided treatment |
|  |  |  |
|  |  | Is there a relationship between the development of cancer and trauma to the breast |
|  |  |  |
|  |  | Does trauma to the breast during intercourse have a role in the occurrence of cancer |
|  |  |  |
|  |  | Can a tight bra increase the incidence of breast cancer? |
|  |  |  |
| What is the most commonplace for proximal breast cancer metastases?  ⬜Armpit ⬜neck ⬜ Supraclavicular | | |
|  |  |  |
|  |  |  |
| Where do you prefer to get breast cancer information?  ⬜doctors ⬜friends & family ⬜Social Media ⬜medical websites ⬜Publications and awareness camping’s | | |

| **Breast cancer common signs and symptoms** | | |
| --- | --- | --- |
| **No** | **Yes** |  |
|  |  | Feeling a mass in the breast |
|  |  |  |
|  |  | Feeling a mass in the armpit |
|  |  |  |
|  |  | Changes in pigmentation |
|  |  |  |
|  |  | Nipple discharge (other than breast milk) |
|  |  |  |
|  |  | Nipple retraction (turning inward) |
|  |  |  |
|  |  | Change in nipple position |
|  |  |  |
|  |  | Change in breast symmetry |
|  |  |  |
|  |  | Ulcers |
|  |  |  |
|  |  | Scaling or thickening of the nipple or breast skin. |
|  |  |  |
|  |  | Redness of the nipple or breast skin. |
|  |  |  |
|  |  | Skin rash on the breast |
|  |  |  |
|  |  | Pain in the armpit |
|  |  |  |
|  |  | Nipple or breast pain |
|  |  |  |

| **Breast cancer risk factors** | | |
| --- | --- | --- |
| **No** | **Yes** |  |
|  |  | Early puberty |
|  |  |  |
|  |  | Late menopause |
|  |  |  |
|  |  | Oral contraceptives |
|  |  |  |
|  |  | Postmenopausal estrogens |
|  |  |  |
|  |  | history of trauma on the breast |
|  |  |  |
|  |  | History of benign tumors |
|  |  |  |
|  |  | History of Inflammatory disease of the breast |
|  |  |  |
|  |  | Alcoholic |
|  |  |  |
|  |  | Smoking |
|  |  |  |
|  |  | Aging |
|  |  |  |
|  |  | Weight gain (BMI> 25) |
|  |  |  |
